# Optimizing methods to detect colonization with fluoroquinolone-resistant viridans group streptococci prior to hematopoietic cell transplantation

**DOI:** 10.1128/jcm.00687-26

**Published:** 2026-08-21

**Authors:** Supram Hosuru Subramanya, Lee S. Gottesdiener, Jamie Marino, Juliet N. Barker, Tsiporah Shore, Rosemary Soave, Markus Plate, John R. Lee, Barry N. Kreiswirth, Laura B. Goodman, Casey L. Cazer, Lars F. Westblade, Michael J. Satlin

**Affiliations:** 1 Department of Pathology and Laboratory Medicine, Weill Cornell Medicine 12295, , New York, New York, USA; 2 Division of Infectious Diseases, Department of Medicine, Weill Cornell Medicine 12295, , New York, New York, USA; 3 Division of Hematology and Oncology, Department of Medicine, Weill Cornell Medicine 12295, , New York, New York, USA; 4 Division of Nephrology and Hypertension, Department of Medicine, Weill Cornell Medicine 12295, , New York, New York, USA; 5 Renal-Electrolyte and Hypertension Division, University of Pennsylvania Perelman School of Medicine 14640, , Philadelphia, Pennsylvania, USA; 6 Center for Discovery and Innovation, Hackensack Meridian Health 721734, Nutley, New Jersey, USA; 7 Department of Public and Ecosystem Health, College of Veterinary Medicine, Cornell University 5922, Ithaca, New York, USA; 8 Department of Clinical Sciences, College of Veterinary Medicine, Cornell University 5922, Ithaca, New York, USA; Children's Hospital Los Angeles, Los Angeles, California, USA

**Keywords:** neutropenia, fluoroquinolone resistance, diagnostics, colonization, viridans streptococci

## Abstract

Patients undergoing hematopoietic cell transplantation (HCT) who receive fluoroquinolone prophylaxis are at high risk for bacteremia due to fluoroquinolone-resistant viridans group streptococci (FQ-R VGS), particularly *Streptococcus mitis*. Detecting colonization with FQ-R VGS may identify neutropenic patients at risk for streptococcal bacteremia during FQ prophylaxis, but the optimal specimen screening site and microbiologic screening method are unknown. We therefore simultaneously collected buccal, oropharyngeal, and perianal swabs from patients upon admission for HCT, and performed selective culture with both direct plating and broth enrichment. FQ-R VGS were detected in 62 (90%) of 69 participants. Direct plating resulted in a positive percent agreement (PPA) of 79% (95% confidence interval [CI]: 67%–88%) for detecting FQ-R VGS from buccal swabs, and a PPA of 86% (95% CI: 74%–93%) from oropharyngeal swabs compared to detection by any specimen or method. Broth enrichment yielded a PPA of 97% (95% CI: 89%–100%) for both buccal and oropharyngeal swabs. Perianal swabs had low PPA. For detecting FQ-R *S. mitis* group, the direct plating PPA was 74% (95% CI: 60%–85%) for buccal and 72% (95% CI: 58%–84%) for oropharyngeal swabs, while the broth enrichment PPA was 82% (95% CI: 69%–91%) and 58% (95% CI: 43%–72%), respectively. Combining direct plating and broth enrichment of buccal swabs had a PPA of 96% (95% CI: 86%–100%) for detecting FQ-R *S. mitis* group. Buccal swabs are a reliable specimen to detect FQ-R VGS colonization when using broth enrichment, and combining direct plating and broth enrichment may optimize detection of FQ-R *S. mitis* group.

**IMPORTANCE:**

Viridans group streptococci (VGS) are important causes of bloodstream infections in patients with cancer undergoing hematopoietic cell transplantation. These infections frequently occur despite using prophylactic fluoroquinolone antibiotics, suggesting that some patients are colonized with fluoroquinolone-resistant VGS (FQ-R VGS). A test that reliably detects carriage of FQ-R VGS might help identify patients at highest risk of streptococcal infection, but the optimal methods to detect FQ-R VGS colonization were previously unknown. In this study, we compared different body sites and culture methods for detecting colonization with FQ-R VGS. We found that buccal (cheek) swabs reliably detected these resistant bacteria, including members of the *Streptococcus mitis* group, and adding a broth enrichment step further improved detection from buccal samples. These findings support buccal sampling with broth enrichment as a practical approach for detecting colonization with FQ-R VGS and establish a foundation for future studies to define the clinical significance of FQ-R VGS colonization.

## INTRODUCTION

Patients with hematologic malignancies who undergo hematopoietic cell transplantation (HCT) are at high risk for bacteremia due to viridans group streptococci (VGS) in the setting of chemotherapy-induced mucositis (1–3). The route of infection is typically translocation of colonizing organisms from the oral and gut mucosa into the bloodstream. The *Streptococcus mitis* group in particular is one of the most important groups of VGS responsible for infections during neutropenia (2–4). VGS bacteremia frequently occurs in this population despite the use of fluoroquinolone (FQ) prophylaxis (5–9). In a multicenter study of patients with fever and neutropenia, most of whom were receiving FQ prophylaxis, VGS were the most common causes of bacteremia, and most VGS isolates were FQ-resistant (FQ-R) (5). Screening for colonization with FQ-R VGS is not a common clinical practice. However, given how frequently HCT recipients become infected with FQ-R VGS, there could be a role in screening patients to determine if they are colonized with FQ-R VGS at mucosal body sites. However, the optimal specimen sites to use to detect colonization with these organisms and the optimal microbiologic methods to use for screening in this population are unknown.

Previous studies assessing FQ-R VGS colonization utilized oropharyngeal swabs (10, 11). However, buccal swabs, which are more comfortable for patients, would be an easier specimen to collect and have been used in microbiome analyses to detect VGS (12). Perianal swabs are collected in some institutions to screen for colonization with resistant gram-negative bacteria and vancomycin-resistant enterococci in patients with hematologic malignancies (13, 14), but it is not clear whether this site could also be useful to screen for colonization with FQ-R VGS. Furthermore, it is not known whether a selective broth enrichment step increases the yield of detecting FQ-R VGS. It is also unknown which methods are ideal for detecting colonization with FQ-R *S. mitis* group, which is the most clinically important group of VGS in this population.

To address these knowledge gaps, we initiated a prospective study to determine the optimal specimen sites and screening methods to detect colonization with FQ-R VGS among patients undergoing HCT at our center. We evaluated culture-based screening methods for FQ-R VGS using swabs from three different sites (buccal, oropharyngeal, and perianal), using both direct plating and broth enrichment of specimens.

## MATERIALS AND METHODS

### Study population

We conducted a prospective observational study at a quaternary care academic medical center in New York City from June 2024 to December 2025. Patients were eligible for inclusion if they were at least 18 years old, admitted to the hospital for HCT, and were scheduled to receive levofloxacin (LVX) prophylaxis during neutropenia. Patients were excluded if they were receiving broad-spectrum antimicrobials expected to have *in vitro* activity against VGS (e.g., vancomycin, piperacillin-tazobactam, and cefepime) within 48 h of enrollment or had already initiated their inpatient LVX prophylaxis regimen at the time of enrollment. Information on malignancy, comorbidities, antibiotic history, and infection history was extracted from electronic health records. Demographic information and history of prior travel were obtained by participant self-report. Written informed consent was obtained from all study participants.

### Specimen collection

One buccal, oropharyngeal, and perianal swab was collected from study participants within 10 days prior to HCT using flocked swabs in Liquid Amies (ESwab; Copan Diagnostics, Murrieta, CA, USA). Buccal swabs were collected by sampling the buccal mucosa bilaterally with five swab rotations on each side. Oropharyngeal swabs were collected by sampling the posterior pharynx and at least one tonsil for up to 2 seconds, as tolerated by the participant. Perianal swabs were self-collected by participants or by nursing staff with instructions to rotate the swab around the external perianal area three times for approximately 15 s. If a perianal swab was previously collected for routine clinical care as part of an institutional protocol to screen for resistant enteric bacteria, then the remnant from this swab was used instead of collecting an additional perianal swab.

### Microbiologic methods

**Fig 1:**
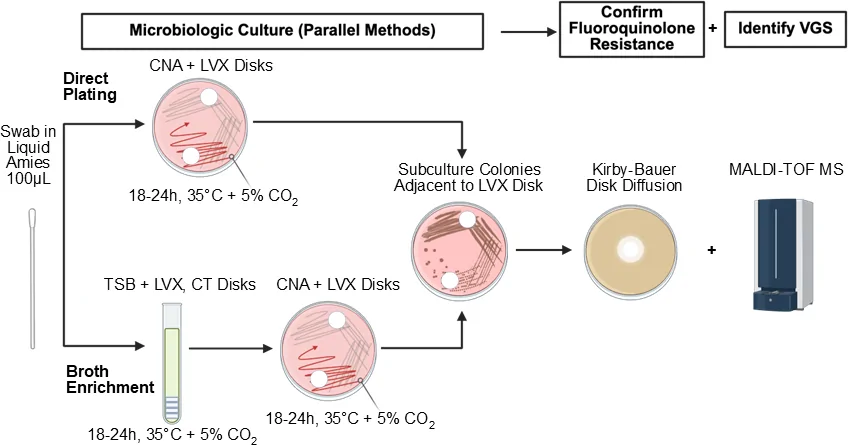
Microbiologic workflow for swab specimens from all sites to isolate and identify colonizing FQ-R VGS strains. Figure created with BioRender.com. CNA, Columbia nalidixic acid; CT, colistin; FQ, fluoroquinolone; LVX, levofloxacin; MALDI-TOF MS, matrix-assisted laser desorption ionization–time of flight mass spectrometry; TSB, tryptic soy broth; VGS, viridans group streptococci.

Buccal, oropharyngeal, and perianal swabs were analyzed within 48 h of collection and were transported and stored at room temperature prior to culture. Specimens underwent selective culture by two methods in parallel: direct plating and broth enrichment (Fig. 1). For direct plating, 100 μL of vortexed Liquid Amies was inoculated onto Columbia nalidixic acid (CNA) agar (Hardy Diagnostics, Santa Maria, CA, USA) with 5 μg LVX disks (Hardy Diagnostics) placed between the first and second quadrants and between the third and fourth quadrants of the agar plate. Plates were incubated for 18–24 h at 35°C with 5% carbon dioxide. For broth enrichment, 100 μL of vortexed Liquid Amies was inoculated into 5 mL of tryptic soy broth (TSB, Becton, Dickinson and Company [BD], Franklin Lakes, NJ, USA) with two 5 μg LVX disks and two 10 μg colistin disks (Hardy Diagnostics), resulting in an enrichment broth with 2 μg/mL of LVX and 4 μg/mL of colistin. These concentrations reflect the Clinical and Laboratory Standards Institute (CLSI) susceptible breakpoint for LVX and VGS and the resistant breakpoint for colistin and Enterobacterales, respectively (15). Broth was incubated for 18–24 h at 35°C with 5% carbon dioxide, after which 100 μL of broth was plated onto CNA agar with LVX disks using the same methodology as direct plating, and incubated in the same fashion. For perianal swabs, 100 μL of vortexed Liquid Amies was also inoculated onto tryptic soy agar (TSA) with 5% sheep blood (BD) as a quality control measure to ensure bacterial growth, verifying mucosal contact during swab collection.

Following incubation of CNA agar with direct plating or broth enrichment methods, plates were examined for growth. Several representative colonies of each distinct morphology consistent with VGS (e.g., alpha hemolysis) that grew within an approximately 20 mm zone diameter around the LVX disk (including disk diameter) were inoculated onto TSA with 5% sheep blood (BD) with a 5 μg LVX disk between the first and second quadrants for isolation of pure colonies growing within a 20 mm zone diameter. A zone diameter was selected that was larger than the LVX disk diffusion susceptibility breakpoint (≥17 mm) to ensure that no FQ-R VGS isolates were missed, given that the medium and inoculum used were not standardized for antimicrobial susceptibility testing (15). FQ resistance for all isolates was then confirmed by disk diffusion using LVX disks on Mueller-Hinton agar with 5% sheep blood (BD), using CLSI interpretive criteria to define resistance (zone diameter ≤13 mm) (15). Only isolates with confirmed resistance to LVX were considered FQ-R.

FQ-R isolates were identified by matrix-assisted laser desorption/ionization time-of-flight mass spectrometry (MALDI-TOF MS) using a United States Food and Drug Administration-cleared reference database (Bruker Daltonics, Billerica, MA, USA). Only isolates identified as VGS by MALDI-TOF MS were included, and identification was made to the species level if possible, or to the group level if the database was unable to identify the exact species (e.g., *S. mitis* group) (16). Optochin disk diffusion was performed at 35°C in ambient air using optochin disks (BD BBL Taxo P Discs, BD) on TSA with 5% sheep blood agar to distinguish *Streptococcus pneumoniae* or *Streptococcus pseudopneumoniae* from other VGS members. *Streptococcus pneumoniae* and *S. pseudopneumoniae* were not included in this analysis due to their distinct clinical characteristics compared to other VGS.

### Statistical methods

For each body site and screening method, positive percent agreement (PPA) was determined with exact binomial 95% confidence intervals (CIs) based on the number of positive tests for that method compared to a reference of the composite of culture positivity from any method or body site. Concordance between each method was calculated with exact binomial 95% CIs based on the frequency that each method produced the same result. Statistical tests were performed in R version 4.4.1.

## RESULTS

We enrolled 69 participants who were admitted for HCT and had specimens collected prior to initiating LVX prophylaxis. The median age of the cohort was 63 years (interquartile range [IQR] = 50–69); 48% were female, 62% identified as White, and 14% identified as Hispanic (Table 1). The most common underlying malignancy was acute leukemia (55%), and 88% of patients were admitted for allogeneic HCT. Forty-nine percent of patients had been hospitalized in the three months prior to enrollment, and 58% had received a FQ in the prior 3 months. Swab collection occurred at a median of 7 days (IQR = 6–7) prior to HCT.

**TABLE 1:** Characteristics of 69 participants undergoing hematopoietic cell transplantation enrolled in the study^*b*^

Characteristic	No. (%) or median (IQR^*a*^)
Age, years, median (IQR)	63 (50–69)
Female sex	33 (48)
Race	
White	43 (62)
Black	13 (19)
Asian	9 (13)
Other	4 (6)
Hispanic ethnicity	10 (14)
Charlson comorbidity index, median (IQR)	4 (3–5)
Underlying malignancy	
Acute leukemia	38 (55)
Myelodysplastic syndrome	15 (22)
Lymphoma	14 (20)
Myeloproliferative neoplasm	11 (16)
Multiple myeloma	1 (1)
Other	4 (6)
Complete remission at time of transplant	47 (68)
Donor type	
Autologous	8 (12)
Double umbilical cord blood	16 (23)
Haploidentical donor	2 (3)
Matched related donor	1 (1)
Matched unrelated donor	27 (40)
Mismatched unrelated donor	15 (22)
Recent healthcare exposures	
Hospitalization in prior 3 months	34 (49)
Fluoroquinolone use in prior 3 months	40 (58)
International travel in prior 6 months	15 (22)

^*a*^ Interquartile range.

^*b*^ Patients may have had more than one malignancy, so totals may exceed 100%.

Sixty-two (90%) of the 69 participants were colonized with FQ-R VGS at any body site by either the direct plating or broth enrichment method. FQ-R VGS were identified in 39 (98%) of the 40 patients who had received a FQ in the prior 3 months and in 23 (79%) of the 29 patients who had not received a FQ in the prior 3 months. FQ-R *S. mitis* group isolates were identified in 50 participants, FQ-R *Streptococcus parasanguinis* in 48, FQ-R *Streptococcus salivarius* group in 3, and FQ-R *Streptococcus sanguinis* in 2. When combining all body sites and both culture methods, 377 VGS isolates were recovered within a 20 mm zone of the LVX disk on CNA agar and upon subculture to TSA with 5% sheep blood. Of these isolates, 368 (98%) were resistant to LVX by disk diffusion susceptibility testing. High-confidence MALDI-TOF MS scores were obtained for 343 (93%) of these 368 isolates, allowing reliable species- or group-level identification. Four FQ-R *S. pneumoniae* isolates were identified and not included in this analysis. Results for each individual participant at each site and by each culture method are displayed in Fig. S1.

**Fig 2:**
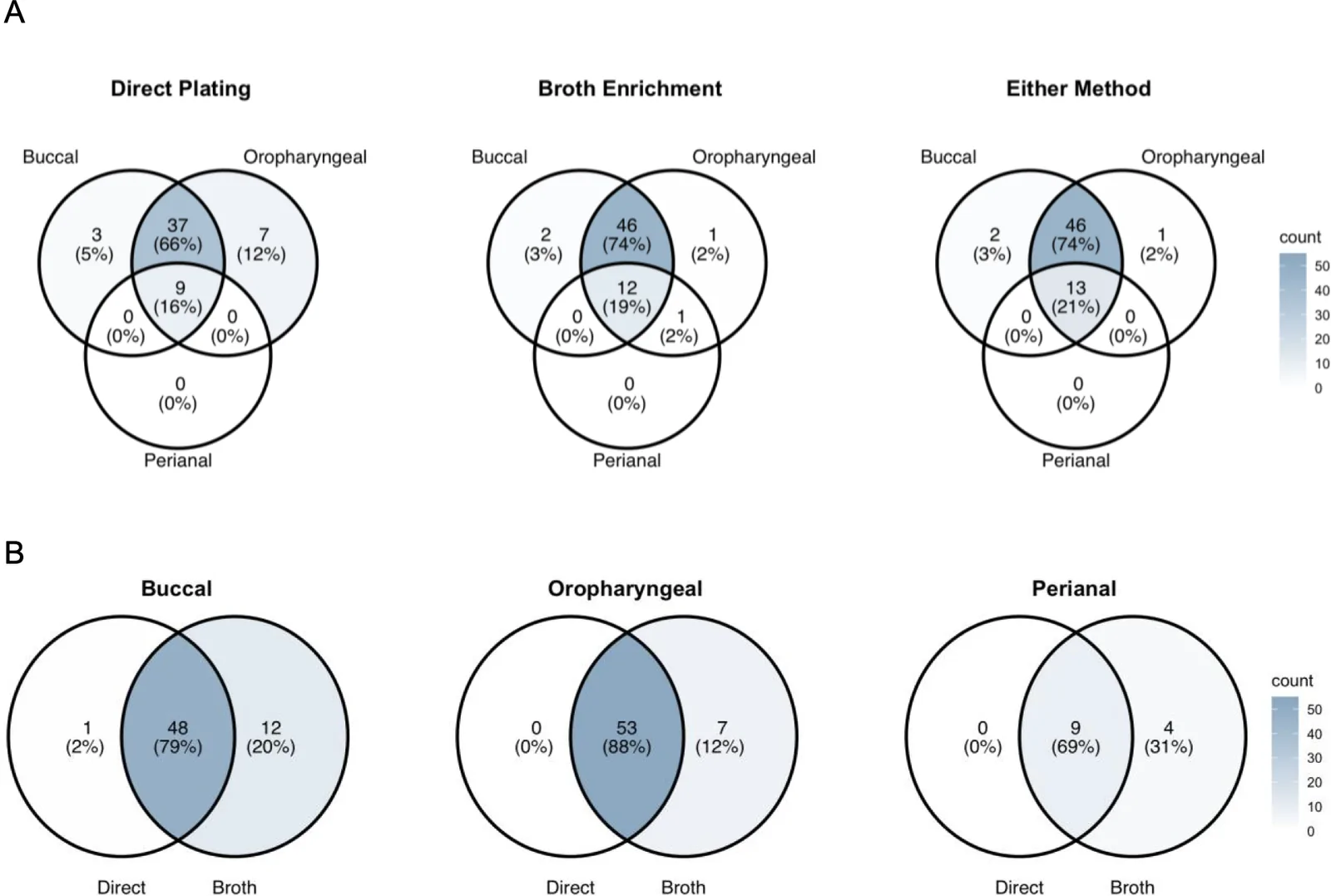
Venn diagrams representing the number of patients with fluoroquinolone-resistant viridans group streptococci detected by (**A**) direct plating, broth enrichment, or a composite of both methods among each body site and (**B**) at each body site among direct plating and/or broth enrichment methods.

A Venn diagram displaying the number of participants with FQ-R VGS detected at each site using direct plating, broth enrichment, or a composite of either method is displayed in Fig. 2. With direct plating, buccal, oropharyngeal, and perianal swabs detected FQ-R VGS in 49, 53, and 9, respectively, of the 62 participants colonized with FQ-R VGS by any site or method. Thus, direct plating resulted in a PPA for buccal swabs of 79% (95% CI: 67%–88%) and a PPA for oropharyngeal swabs of 86% (95% CI: 74%–93%) (Table 2). With broth enrichment, both buccal and oropharyngeal swabs detected FQ-R VGS in 60 of the 62 colonized participants, corresponding to a PPA of 97% (95% CI: 89%–100%) for each. In contrast, perianal swabs detected FQ-R VGS in only 9 participants (15%) with direct plating and 13 (21%) with broth enrichment. Concordance between buccal and oropharyngeal swabs using the direct plating and broth enrichment methods ranged from 81% to 94%, but was lower between these sites and perianal swabs (Table S1A).

**TABLE 2:** Positive percent agreement for detection of FQ-R VGS and FQ-R *S. mitis* group for each site and method^*a*^

Specimen site	Positive percent agreement (95% CI)		
	Direct plating	Broth enrichment	Either method
FQ-R VGS			
Buccal	79 (67–88)	97 (89–100)	98 (91–100)
Oropharyngeal	86 (74–93)	97 (89–100)	97 (89–100)
Perianal	15 (7–26)	21 (12–33)	21 (12–33)
FQ-R *S. mitis* group			
Buccal	74 (60–85)	82 (69–91)	96 (86–100)
Oropharyngeal	72 (58–84)	58 (43–72)	78 (64–89)
Perianal	10 (3–22)	16 (7–29)	16 (7–29)

^*a*^ Data are presented as percentages (95% CI). The reference comparator is detection of the organism(s) by any method or specimen site.

**Fig 3:**
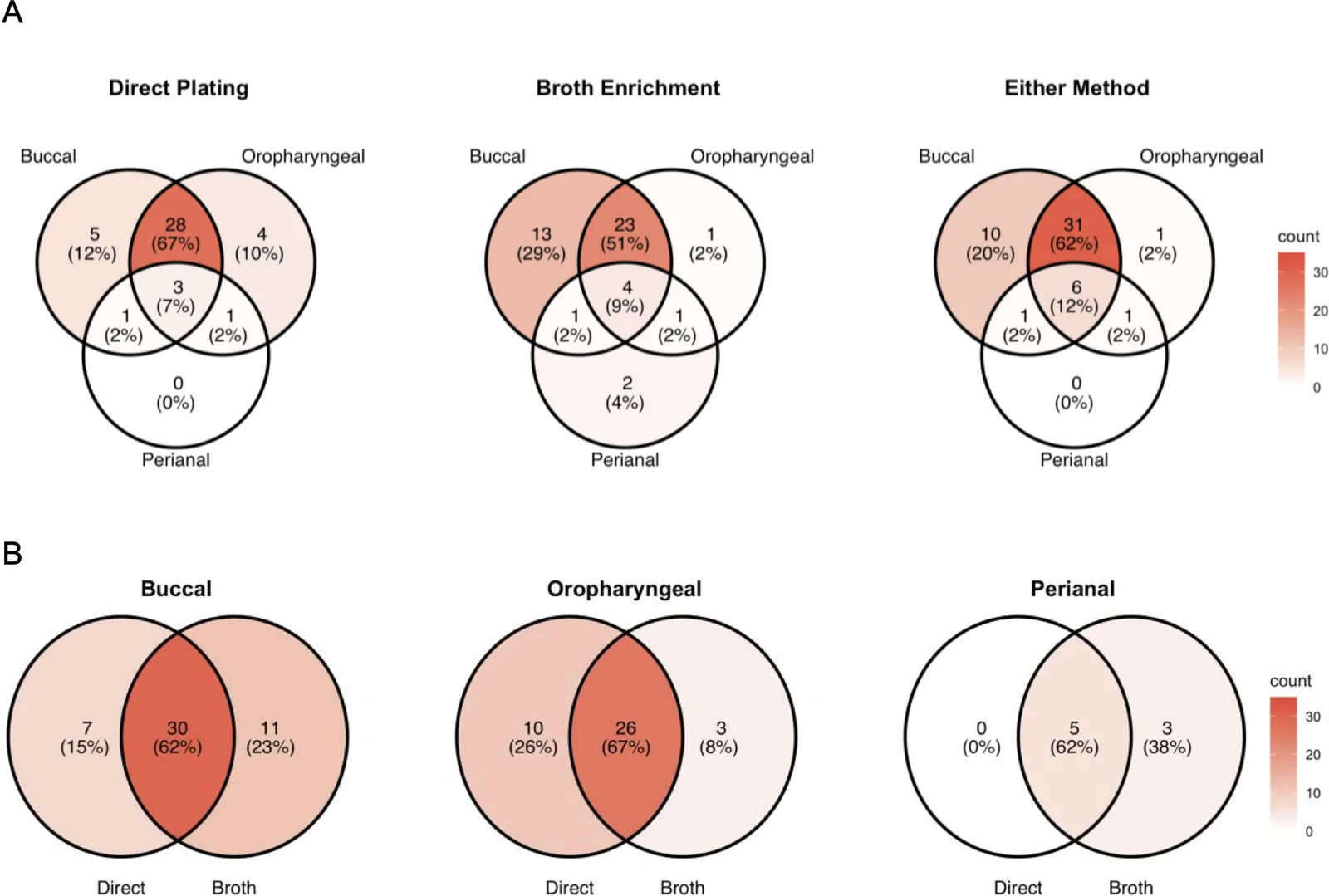
Venn diagrams representing the number of patients with fluoroquinolone-resistant *S. mitis* group detected by (**A**) direct plating, broth enrichment, or a composite of both among each body site and (**B**) at each body site among direct plating and/or broth enrichment methods.

For the 50 participants colonized with FQ-R *S. mitis* group, a Venn diagram displaying the number of participants with FQ-R *S. mitis* group detected by each method is displayed in Fig. 3. For the FQ-R *S. mitis* group, direct plating resulted in a PPA for buccal swabs of 74% (95% CI: 60%–85%) and a PPA for oropharyngeal swabs of 72% (95% CI: 58%–84%) (Table 2). Broth enrichment had a higher PPA for buccal swabs of 82% (95% CI: 69%–91%), but a lower PPA for oropharyngeal swabs at 58% (95% CI: 43%–72%). A composite of either direct plating or broth enrichment resulted in a PPA for buccal swabs of 96% (95% CI: 86%–100%), but for oropharyngeal swabs of only 78% (95% CI: 64%–89%). Concordance between individual buccal and oropharyngeal methods for detection of FQ-R *S. mitis* group ranged from 74% to 84% (Table S1B).

## DISCUSSION

FQ-R VGS are common bloodstream pathogens in neutropenic patients receiving FQ prophylaxis, but the optimal methods to detect colonization with these organisms were previously unknown. In this prospective investigation of patients undergoing HCT, buccal and oropharyngeal swabs demonstrated a PPA of 79% and 86%, respectively, for detecting FQ-R VGS with direct plating. These PPAs increased to 97% for each specimen site when broth enrichment was used prior to plating.

While buccal and oropharyngeal swabs demonstrated relatively high yield for detecting FQ-R VGS, perianal swabs had low yield. Although past studies investigating FQ-R VGS have utilized oropharyngeal swabs, results from this study support the use of buccal swabs for screening, which are less invasive and do not produce an uncomfortable gagging sensation for patients. Although perianal swabs are already collected in some centers to screen for colonization with multidrug-resistant gram-negative bacteria (13, 14), this study shows their yield to detect FQ-R VGS is limited.

There was a higher PPA for detecting FQ-R VGS using a broth enrichment culture for buccal and oropharyngeal swabs compared to direct plating, which could potentially justify the additional time and cost for this additional step in the laboratory. The use of broth enrichment to enhance the yield of VGS is consistent with prior literature assessing VGS recovery from different body sites (17). However, the impact of broth enrichment may be more nuanced when screening for specific VGS species. For FQ-R *S. mitis* group specifically, broth enrichment resulted in only a modest increase in PPA for buccal swabs, and a surprisingly lower PPA for oropharyngeal swabs. Combining direct plating and broth enrichment resulted in the highest PPA to detect FQ-R *S. mitis* group, particularly for buccal swabs. We hypothesize that the higher yield of buccal swabs compared to oropharyngeal swabs for detecting colonization with FQ-R *S. mitis* group may reflect the known predilection of *S. mitis* for the buccal mucosa (18, 19). It is not clear why broth enrichment of oropharyngeal swabs had a lower yield for FQ-R *S. mitis* group compared to direct plating. However, we hypothesize that this finding may be due to the FQ-R *S. mitis* group being outcompeted during broth enrichment by other FQ-R organisms, such as staphylococci or other VGS species like *S. parasanguinis*. This concept of other organisms outcompeting the pathogen of interest during broth enrichment has been observed for *Listeria monocytogenes* (20). This decrease in yield with broth enrichment culture compared to direct plating was not observed for buccal swabs, suggesting growth of competing organisms was less of a factor for this specimen type. However, the yield of recovery of FQ-R *S. mitis* group from buccal swabs increased from 82% with broth enrichment alone to 96% when both methods were used. Therefore, if identification of FQ-R *S. mitis* group is important, it may be optimal to perform culture of buccal swabs with both direct plating and with a broth enrichment step.

An unexpected finding from this study was that 90% of participants were colonized with FQ-R VGS prior to their transplant and prior to initiating FQ prophylaxis, and 72% were colonized with FQ-R *S. mitis* group. This may explain why breakthrough bloodstream infections due to VGS are so common in HCT recipients in the setting of FQ prophylaxis (5, 6). The reasons for this high colonization prevalence are unclear, but more than one-half of participants had received FQs in the 3 months prior to their study participation. Discrimination of colonizing VGS by specific clinically relevant groups such as the *S. mitis* group, the most common cause of bacteremia in this population, may have further clinical significance (2–4, 16). Additional studies are needed to better understand the colonization prevalence of FQ-R VGS in patients undergoing HCT at other centers and in other neutropenic populations.

This study has several limitations. First, LVX was used in this study to define FQ-R because it is the most commonly used FQ for prophylaxis in neutropenic patients and has a CLSI interpretive breakpoint (15, 21). However, resistance to other FQs may be variable. Second, the culture-based methods used rely on discriminating between morphologically distinct colonies on agar plates, and therefore could be prone to omitting isolates of different VGS species that are unable to be distinguished from each other by visual inspection. Third, this study used specific culture media, including CNA agar and TSB; results may not be generalizable to other media or enrichment methods. Fourth, MALDI-TOF MS has limited ability to identify certain VGS to the species level, particularly among the *S. mitis* group, resulting in less precise identification for these clinically important organisms. Finally, we did not perform quantitative cultures to evaluate the burden of colonization with FQ-R VGS. Future research could assess associations between FQ-R VGS quantitative burden and VGS bacteremia risk.

Based on our results, we believe that buccal swabs are an optimal specimen site for screening for colonization with FQ-R VGS based on their test performance and ease of collection. Moreover, simultaneous broth enrichment and direct plating may optimize the yield of cultures of these buccal swabs for FQ-R VGS. By establishing the performance of different specimen sites and microbiologic methods to detect colonization with FQ-R VGS, this study creates a foundation for future clinical investigations into the risk factors and clinical significance of colonization with FQ-R VGS, which are common causes of bacteremia among neutropenic patients receiving FQ prophylaxis. Clinical studies to examine the prevalence of these organisms in different populations and the association between colonization and infection are ongoing. Future studies may be able to leverage an understanding of detecting FQ-R VGS colonization to prevent VGS infections more effectively in neutropenic patients.

## SUPPLEMENTAL MATERIAL

Fig. S1 and Table S1 jcm.00687-26-s0001.docx Fig. S1: Individual-level FQ-R VGS and FQ-R *S. mitis* group positivity at each site and by each method, ordered by number of total positive results. Table S1: Concordance between tests for detection of (A) FQ-R VGS and (B) FQ-R *S. mitis* group.

ASM does not own the copyrights to Supplemental Material that may be linked to, or accessed through, an article. The authors have granted ASM a non-exclusive, world-wide license to publish the Supplemental Material files. Please contact the corresponding author directly for reuse.
