# Supplementary material for: Optimizing methods to detect colonization with fluoroquinolone-resistant viridans group streptococci prior to hematopoietic cell transplantation
Source: J Clin Microbiol. 2026 Aug 21;64(9):e00687-26. doi: 10.1128/jcm.00687-26 (PMC13556186; doi:10.1128/jcm.00687-26)
Supplement: Supplementary file 1 [file jcm.00687-26-s0001.docx]

**SUPPLEMENTARY MATERIALS**

**Table of Contents	Page**

| Table of Contents page | 1 |
| --- | --- |
| Supplemental Figure 1. Individual-level FQ-R VGS and FQ-R *S. mitis* group positivity at each site and by each method, ordered by number of total positive results. | 2 |
| Supplemental Table 1. Concordance between tests for detection of A) FQ-R VGS and B) FQ-R *S. mitis* group | 3-4 |

 **Supplemental Figure 1.** Individual-level FQ-R VGS and FQ-R *S. mitis* group positivity at each site and by each method, ordered by number of total positive results. Participants with prior FQ use within the 3 months noted with an asterisk.

**Supplemental Table 1.** Concordance between tests for detection of A) FQ-R VGS and B) FQ-R *S. mitis* group

A)

|  | Buccal  Direct Plating | Buccal  Broth Enrichment | Oropharyngeal  Direct Plating | Oropharyngeal  Broth Enrichment | Perianal  Direct Plating | Perianal  Broth Enrichment |
| --- | --- | --- | --- | --- | --- | --- |
| Buccal  Direct Plating |  |  |  |  |  |  |
| Buccal  Broth Enrichment | 81 (70-90) |  |  |  |  |  |
| Oropharyngeal  Direct Plating | 86 (75-93) | 87 (77-94) |  |  |  |  |
| Oropharyngeal  Broth Enrichment | 84 (73-92) | 94 (86-98) | 90 (80-96) |  |  |  |
| Perianal  Direct Plating | 42 (30-55) | 23 (14-35) | 36 (25-49) | 26 (16-38) |  |  |
| Perianal  Broth Enrichment | 48 (36-60) | 29 (19-41) | 42 (30-55) | 32 (21-44) | 94 (86-98) |  |

Data are presented as % (95% confidence interval).

B)

|  | Buccal  Direct Plating | Buccal  Broth Enrichment | Oropharyngeal  Direct Plating | Oropharyngeal  Broth Enrichment | Perianal Direct Plating | Perianal  Broth Enrichment |
| --- | --- | --- | --- | --- | --- | --- |
| Buccal  Direct Plating |  |  |  |  |  |  |
| Buccal  Broth Enrichment | 74 (62-84) |  |  |  |  |  |
| Oropharyngeal  Direct Plating | 84 (73-92) | 75 (64-85) |  |  |  |  |
| Oropharyngeal  Broth Enrichment | 74 (62-84) | 77 (65-86) | 81 (70-90) |  |  |  |
| Perianal  Direct Plating | 51 (38-63) | 45 (33-57) | 52 (40-64) | 59 (47-71) |  |  |
| Perianal  Broth Enrichment | 55 (43-67) | 44 (32-56) | 57 (44-68) | 61 (48-72) | 96 (88-99) |  |

Data are presented as % (95% confidence interval).
